# Outcomes of Patients With Autosomal Dominant Polycystic Kidney Disease Prescribed SGLT2 Inhibitors in British Columbia: A Single-Arm Retrospective Cohort Study

**DOI:** 10.1177/20543581251404101

**Published:** 2025-12-12

**Authors:** Alessandro Cau, Mark Elliott, Adeera Levin, Charith Karunarathna, Alexandra Romann, Ognjenka Djurdjev, Mohammad Atiquzzaman, Micheli Bevilacqua

**Affiliations:** 1 University of British Columbia, Vancouver, Canada; 2 BC Renal Agency, Vancouver, Canada

**Keywords:** autosomal dominant polycystic kidney disease, chronic kidney disease, acute kidney injury, clinical research, British Columbia

## Abstract

**Background:**

Autosomal dominant polycystic kidney disease (ADPKD) is the fourth leading cause of kidney failure in Canada and internationally. To date, patients with ADPKD have been excluded from trials of sodium-glucose cotransporter type 2 inhibitors (SGLT2i), which have been demonstrated to positively influence a wide range of kidney outcomes across the spectrum of chronic kidney disease (CKD). This exclusion was primarily due to theoretic safety concerns, particularly hastening disease progression due to vasopressin stimulation. As a result, there is a paucity of data on SGLT2i use among patients with ADPKD.

**Objectives:**

To estimate the risk of kidney dysfunction with SGLT2i treatment among patients with ADPKD.

**Design:**

Single-arm retrospective cohort study.

**Setting and patients:**

Adult patients (≥18 years old) with CKD with a primary diagnosis of ADPKD in British Columbia, Canada who had been exposed to any drug formulation containing empagliflozin, dapagliflozin or canagliflozin.

**Methods and measurements:**

We retrieved existing data from the province wide registry of patients with kidney disease and performed manual chart reviews on patients with ADPKD who were prescribed an SGLT2i from January 1, 2014, to December 31, 2024. The primary outcome was acute kidney injury (AKI). Secondary outcomes included eGFR slope before and after SGLT2i initiation, magnitude of “eGFR dip” after starting SGLT2i as well as the incidence of genitourinary (GU) infections requiring hospital admission, emergency room visit and/or outpatient diagnosis and treatment.

**Results:**

We included 17 patients on SGLT2i in our retrospective chart review with a median exposure of 20.89 months. While on an SGLT2i, one (6%) patient met criteria for AKI. Three patients (18%) had an eGFR dip of greater than 10% after starting an SGLT2i. Before SGLT2i initiation, the estimated eGFR slope was −0.2571 mL/min/1.73 m^2^. After initiation, the slope was −0.1435 mL/min/1.73 m^2^ (*P* = .48). Two patients (12%) had documentation of a urinary tract infection, neither of whom required hospitalization, or an emergency department visit.

**Limitations:**

The main limitation was the lack of a comparator group, thereby making it difficult to determine the true risk of AKI in our cohort of patients with ADPKD on SGLT2i. Other limitations include our retrospective study design and small sample size, which limits the generalizability of these results. The median exposure time of our cohort to SGLT2i was only 20.89 months and we had limited eGFR data beyond 2 years post-SGLT2i initiation. We did not have data on total kidney volume of these patients.

**Conclusions:**

In this cohort of 17 patients with ADPKD on SGLT2i, we did not observe any signs of adverse kidney outcomes and only two instances of GU infections occurred, neither requiring emergency visits or hospitalization. More high-quality evidence is needed to determine the safety and efficacy of SGLT2i in this population.

## Abrégé

**Contexte:**

La polykystose rénale autosomique dominante (ADPKD) est la quatrième cause d’insuffisance rénale au Canada et dans le monde. À ce jour, les personnes atteintes d’ADPKD ont été exclues des essais portant sur les inhibiteurs du cotransporteur sodium-glucose de type 2 (iSGLT-2), malgré l’efficacité de ces derniers sur un large éventail de paramètres rénaux dans le spectre de l’insuffisance rénale chronique (IRC). Cette exclusion reposait principalement sur des préoccupations théoriques concernant la sécurité, en particulier du risque d’aggravation de la maladie lié à la stimulation de la vasopressine. Par conséquent, les données sur l’utilisation des iSGLT2 en contexte d’ADPKD sont limitées.

**Objectif:**

Estimer le risque de dysfonctionnement rénal lié à un traitement par iSGLT2 chez les patients atteints d’ADPKD.

**Conception:**

Étude de cohorte rétrospective à bras unique.

**Cadre et sujets de l’étude:**

Des adultes (≥18 ans et plus) de Colombie-Britannique (Canada) atteints d’IRC, dont le diagnostic primaire est l’ADPKD, exposés à une formulation médicamenteuse contenant de l’empagliflozine, de la dapagliflozine ou de la canagliflozine.

**Méthodologie:**

Nous avons extrait les données du registre provincial des patients atteints d’insuffisance rénale, et procédé à une révision manuelle des dossiers des patients atteints d’ADPKD ayant reçu une prescription d’iSGLT2 entre le 1^er^ janvier 2014 et le 31 décembre 2024. Le principal critère de jugement était l’insuffisance rénale aiguë (IRA). Les critères secondaires incluaient la pente du DFGe avant et après l’initiation des iSGLT2, l’ampleur de la « chute du DFGe » observée après l’initiation des iSGLT2, et l’incidence des infections génito-urinaires (GU) nécessitant une hospitalisation, une visite aux urgences et/ou un diagnostic et un traitement en consultation externe.

**Résultats:**

En tout, 17 patients sous iSGLT2 ont été inclus dans l’analyse rétrospective, avec une période médiane d’exposition de 20,89 mois. Pendant le traitement par iSGLT2, un patient (6 %) a présenté un épisode d’IRA répondant aux critères diagnostiques. Trois patients (18 %) ont présenté une baisse du DFGe de plus de 10 % après le début du traitement. La pente du DFGe est passée de - 0,2571 ml/min/1,73 m^2^ avant l’initiation du traitement à - 0,1435 ml/min/1,73 m^2^ (p = 0,48) après. Deux patients (12 %) ont présenté une infection GU documentée, mais aucune n’a nécessité une hospitalisation ou une visite aux urgences.

**Limites:**

L’absence de groupe comparateur constitue la principale limite de cette étude, et rend incertaine l’estimation précise du risque d’IRA chez les patients atteints d’ADPKD sous iSGLT2. La conception rétrospective de l’étude et la faible taille de l’échantillon limitent la généralisabilité des résultats. La durée médiane d’exposition aux iSGLT2 de la cohorte n’était que de 20,89 mois, avec des données limitées sur le DFGe au-delà de deux ans après le début du traitement. Nous ne disposions pas de données sur le volume rénal total (VRT) des patients.

**Conclusion:**

Dans notre cohorte de 17 patients atteints d’ADPKD traités par iSGLT2, aucun événement rénal indésirable n’a été observé. Deux cas d’infections GU ont été recensés, mais aucun n’a nécessité une hospitalisation ou une visite aux urgences. Davantage de données de haute qualité sont requises pour évaluer l’innocuité et l’efficacité des iSGLT2 dans cette population.

## Introduction

Autosomal dominant polycystic kidney disease (ADPKD) is the fourth leading cause of kidney failure in Canada and internationally.^1,2^ The current mainstay of treatment for patients with ADPKD includes blood pressure control with a renin-angiotensin system (RAS) inhibitor, sodium restriction under 2 g per day, and greater than 3 L of water intake per day.^3^ In select high risk patients, tolvaptan can be added which has been shown to slow the rate of increase in total kidney volume (TKV) and the rate of decline in kidney function.^4,5^ Tolvaptan is a vasopressin antagonist, which leads to decreased cyst cell proliferation, fluid secretion, cystogenesis, and renal enlargement.^6^

Sodium-glucose cotransporter type 2 inhibitors (SGLT2i) have been demonstrated in various landmark trials to have protective effects across a spectrum of kidney outcomes in patients with and without diabetes.^7^ Therefore, SGLT2i could represent a novel therapeutic option for patients with ADPKD as well. However, patients with ADPKD have been excluded from these large trials due to concern that treatment with an SGLT2i may theoretically increase vasopressin levels, subsequently resulting in more cyst formation and transepithelial fluid secretion, thus hastening disease progression.^8-10^ For these reasons, recent Kidney Disease Improving Global Outcomes (KDIGO) guidelines on patients with ADPKD have not suggested a role for SGLT2i due to insufficient safety data.^11^ There is also the risk of exacerbating hypovolemia, hypernatremia and acute kidney injury (AKI) in patients already taking RAS inhibitors and tolvaptan. Furthermore, SGLT2i promote glucosuria and have been associated with genital mycotic infections, but not urinary tract infections, in the general population.^12^ Therefore, in patients with ADPKD, for whom data are lacking and who are uniquely at higher overall risk of genitourinary (GU) infections, SGLT2i could further increase this risk.

The current animal data are difficult to interpret as the models do not optimally represent ADPKD in humans, making the translation of these results to humans challenging.^13-16^ In this single arm retrospective cohort study, our objective was to determine if there are observable increases in AKI, GU infections, as well as changes in eGFR slope with SGLT2i treatment among patients with ADPKD.

## Methods

### Setting and Study Design

This study was a single arm retrospective observational cohort study that took place in British Columbia (BC), Canada. Research ethics board approval was obtained for this study (UBC REB H24-00056).

### Reporting Guidelines

We followed the Strengthening the Reporting of Observational Studies in Epidemiology (STROBE) checklist in the preparation of this manuscript^17^ (see Supplemental Material).

### Data Extraction and Management

Study data were retrieved from existing data within the BC province-wide integrated registry and clinical information system for kidney disease, known as PROMIS (Patient Records and Outcome Management Information System). In PROMIS, demographic and other nonlaboratory variables are categorized by their nephrologist and/or renal nurse based on the available clinical data, including imaging and medical chart data. Laboratory results are integrated directly into PROMIS through an automated interface. Hospitalization and emergency department (ED) visit data as well as any positive urine culture results were manually retrieved from patient charts through the province wide electronic medical record. The data were reviewed by two independent reviewers (A.C. and C.K.). Inter-rater reliability was not assessed.

### Study Population

The cohort included all adult chronic kidney disease (CKD) patients with a primary diagnosis of ADPKD with any exposure to SGLT2i from January 1, 2014, to time to maintenance dialysis initiation, kidney transplantation, death, move out of province, lost-to-follow-up or December 31, 2024, whichever came first. In PROMIS, the patient’s cause of CKD is categorized by their nephrologist based on the available clinical data. Given the diagnosis of ADPKD is based mainly on clinical data including family history and imaging, this is the criteria that was used for the most diagnoses. In line with ADPKD guidelines, genetic testing is reserved for atypical cases, de novo cases, and those cases where it is felt to impact clinical management and so the cases in this cohort are not genetically confirmed. The inclusion criteria included patients greater than or equal to 18 years of age with a primary diagnosis of ADPKD and exposure to any drug formulation containing empagliflozin, dapagliflozin and/or canagliflozin. Patients were excluded if they had any history of dialysis or kidney transplant prior to exposure to SGLT2i.

### Outcomes of Interest

The primary outcomes of interest were a) acute kidney injury (AKI) b) the magnitude of “eGFR dip” when initiating SGLT2i, as defined by percentage change between preexposure eGFR and next eGFR after SGLT2i initiation, and c) the presence of GU infections requiring hospital admission, emergency room visit and/or outpatient diagnosis and treatment. AKI was defined according to KDIGO serum creatinine-based criteria. Other variables of interest included baseline demographic and clinical characteristics of the study cohorts.

### Statistical Analysis

We fit a piecewise linear mixed-effects model to estimate eGFR slopes before and after SGLT2i initiation. The primary comparison was the difference in slopes (After − Before), tested 2-sided at α = 0.05. We report the contrast estimate and p-value. Due to the small sample size, findings are exploratory. To minimize conflation of the acute dip with post-initiation, we centered all eGFR values at the first postinitiation eGFR and modeled the eGFR change relative to the first post initiation eGFR as the outcome in the piecewise linear mixed-effects model with separate time terms pre- and post-initiation periods.

### Missing Data

Missing data were not imputed due to small sample size which limited our ability to do so. In terms of outliers, data were retrieved from the PROMIS database, which has a preexisting data dictionary including filtering out of outlier data that are suspected to be erroneous, so those data have already been removed prior to data retrieval for this project. Specific to GU infections, if those data were missing from PROMIS, they were retrieved from existing hospital records in the province-wide electronic medical record.

## Results

### Baseline Characteristics

Table 1 shows the characteristics of 17 patients with ADPKD included in the present study. There were more male patients (10/17, 58%) than female patients (7/17, 42%). The mean age and eGFR of the cohort at SGLT2i initiation was 66.1 years and 40.9 mL/min/1.73 m^2^, respectively. The median exposure time of our cohort to SGLT2i was 20.89 months. The most prevalent comorbidities include diabetes mellitus (10/15), coronary artery disease (CAD, 5/15), and congestive heart failure (3/15). We were unable to retrieve comorbidity data for two patients. Regarding TKV measurements, 8/17 pts have TKV recorded in PROMIS. Two of them have TKV data after SGLT2i initiation, whereas six patients have TKV data before SGLT2i initiation. No patients have TKV data both before and after the initiation. For patients with TKV data prior to initiation, TKV data were collected 0.7 to 6.4 years prior to initiation. For patients with TKV data post initiation, TKV measurements were collected between 0.14 and 1.65 years after SGLT2i initiation. Tolvaptan was prescribed for 2/13 (15%) patients, and 13/13 (100%) patients were on an RAS inhibitor. We were unable to retrieve data on concurrent medication use for four patients. Regarding the choice of SGLT2i, 8/17 (47%) were on dapagliflozin, whereas the remainder were on empagliflozin (9/17, 53%).

**Table 1.** Characteristics of included patients.

Patient characteristics at SGLT2i initiation	N (%)
N	17
Mean Age at SGLT2i initiation (y)	66.1
Male Sex	10 (58%)
Mean eGFR at SGLT2i (mL/min/1.73m^2^)	40.9
TKV Data	8 (47%)
Comorbidities	
Diabetes	10/15^a^ (67%)
Heart failure	3/15^a^ (20%)
Coronary artery disease	5/15^a^ (33%)
Concurrent Medication	
RAS inhibitor	13/13 (100%)
Diuretic	6/13^a^ (46%)
Tolvaptan	2/13^a^ (15%)
SGLT2 inhibitor	
Dapagliflozin	8/17 (47%)
Empagliflozin	9/17 (53%)

SGLT2i: sodium-glucose cotransporter 2 inhibitor; eGFR: estimated glomerular filtration rate; RAS: renin-angiotensin system; TKV: total kidney volume.

a Indicates missing data.

### Development of Acute Kidney Injury

While on an SGLT2i, only one of 17 (6%) patients met criteria for AKI.

### EGFR Slope Before and After SGLT2i Initiation

**Figure 1.**
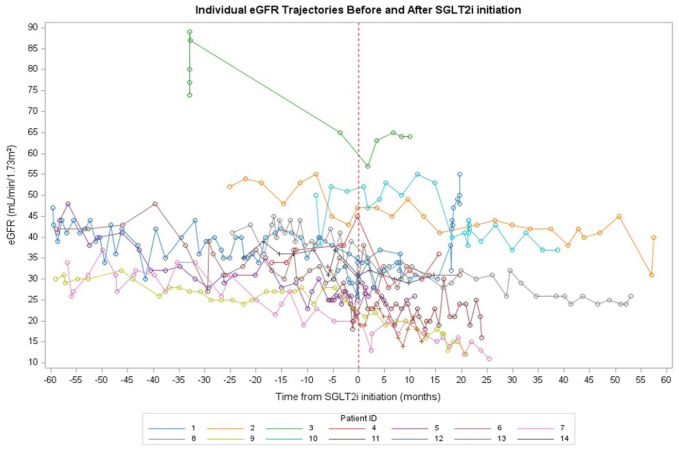
eGFR slope before and after SGLT2i initiation (n = 14).

We were only able to calculate eGFR slopes for 14/17 patients as we did not have sufficient eGFR data pre-SGLT2i and post-SGLT2i initiation for three patients. Before SGLT2i initiation, the estimated eGFR slope was −0.2571 mL/min/1.73 m^2^ (Figure 1). After initiation, the slope was −0.1435 mL/min/1.73 m^2^. The difference in slopes (After – Before) was 0.1136 mL/min/1.73 m^2^, indicating a slower decline in kidney function after treatment initiation; however, this difference was not statistically significant (*P* = .48) (Figure 1).

### Development of eGFR Dip After Starting SGLT2i

**Figure 2.**
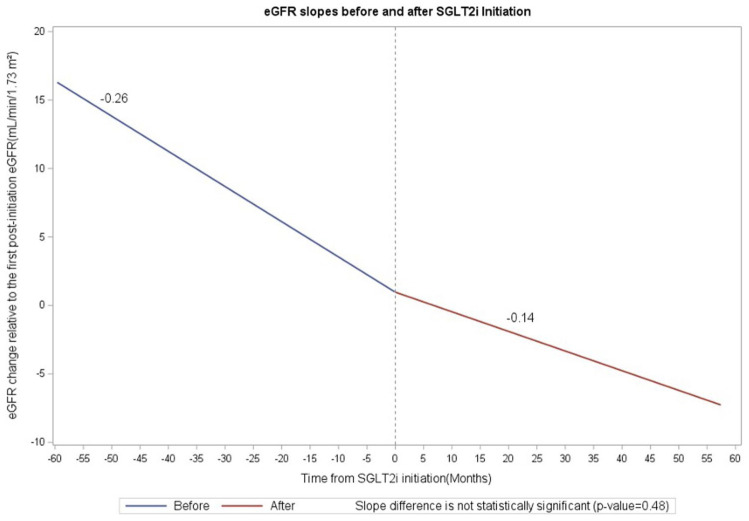
eGFR trajectories of included patients (n = 14).

Of 17 patients, nine did not experience an eGFR dip after starting treatment (Figure 2). Of the eight remaining patients, five patients had an eGFR dip of <10% and three patients experienced an eGFR dip of 10% to 30%.

### Urinary Tract Infections

While on an SGLT2i, two of 17 (12%) patients had documentation of a urinary tract infection, neither of whom required hospitalization, or an ED visit.

## Discussion

In this study, we describe a cohort of 17 patients with ADPKD prescribed SGLT2i across British Columbia. We observed that in 14 patients for whom we were able to obtain eGFR data pretreatment and post-treatment initiation, there appeared to be no difference in kidney function after starting SGLT2i. There was only 1 patient who developed an AKI and only two instances of GU infections, none of which required hospitalization or ED visit.

There are concerns that SGLT2i use could hasten disease progression in patients with ADPKD by increasing cystogenesis. Hence, patients with ADPKD have been excluded from all major SGLT2i trials to date. In our cohort of patients with ADPKD, SGLT2i use was not associated with significant adverse kidney outcomes. However, there is heterogeneity in published results regarding the efficacy and safety of SGLT2i use among patients with ADPKD. In one case report, dapagliflozin addition to baseline tolvaptan and RAS inhibition slowed the patient’s decline in eGFR, whereas in another instance, dapagliflozin added to a background of RAS inhibition alone appeared to be correlated with worsening eGFR.^18,19^ TKV measurements either remained stable or increased when dapagliflozin was added.

Additionally, we came across several observational studies regarding the use of SGLT2i in patients with ADPKD. Morioka and colleagues published a single arm retrospective case series on 20 patients with ADPKD who were prescribed dapagliflozin for a mean of 102 ± 20 days.^20^ They found that dapagliflozin was associated with a statistically significant reduction in eGFR (*P* < .001). Yoshimoto et al^21^ performed a retrospective case study on 7 patients with ADPKD on dapagliflozin in Tokyo, Japan. They observed patients for a median of 20 months after dapagliflozin initiation. Dapagliflozin appeared to have a statistically significant protective effect on eGFR slope among patients on RAS inhibition (*P* = .04).

It has been well described in large trials that SGLT2i treatment is associated with an eGFR “dip” following initiation of treatment as well as a long-term decrease in the rate of eGFR decline.^7^ As these trials excluded patients with ADPKD, it is unclear whether this same effect occurs in patients with ADPKD. Current observational studies of patients with ADPKD treated with SGLT2i showed that most patients did experience an initial eGFR dip.^20,21^ In our study of 17 patients with ADPKD, however, 14 patients experienced no eGFR dip or an eGFR dip < 10% after starting SGLT2i. Furthermore, only three patients had an eGFR dip of greater than or equal to 10%. This may have been due to our small sample size and differing lab work schedules. Similar to work done by Yoshimoto and colleagues, however, we did demonstrate a nonsignificant decrease in the rate of eGFR decline following SGLT2i treatment initiation. This effect was not observed in the cohort described by Morioka and colleagues, who observed a continued rate of decline in eGFR following SGLT2i initiation.^20^ They postulated that this was due to increased cyst formation, as there was a statistically significant increase in height-adjusted TKV. It has been proposed that the statistically significant increase in height-adjusted TKV following SGLT2i initiation may result from increased cystogenesis through increased glucose absorption via SGLT1.^19^ What remains to be seen is whether the kidney protective effects of SGLT2i can offset the decline in eGFR associated with increased cyst volume among patients with ADPKD.

The main limitation of this study was that we lacked a comparator group without SGLT2i exposure, making it difficult to draw conclusions about the impact of SGLT2i use on our outcomes of interest. Even if we did have a comparator group, it would be unlikely that we would be able to draw any conclusions from the comparisons given our small exposure cohort which would limit the power of any analysis that was completed. The small sample size additionally limits the generalizability of these results. Furthermore, the median exposure time of our cohort to SGLT2i was only 20.89 months and we had limited eGFR data beyond two years post-SGLT2i initiation. Thus, the long-term kidney effects of exposure to SGLT2i in our patients are unknown. Another limitation is that we only had TKV data for a subset of patients and we had no serial TKV data over time, which may be an earlier way to detect any changes in disease progression. Our results, while exploratory, suggest that SGLT2i may be safe to use in patients with ADPKD and, similar to work done by others, inform larger scale studies on this topic.

## Conclusions

In our cohort of patients with ADPKD taking RAS inhibitors, SGLT2i did not result in identifiable adverse kidney outcomes including AKI and GU infections. More high-quality evidence is needed to determine the safety and efficacy of SGLT2i in this population.

## Supplemental Material

sj-pdf-1-cjk-10.1177_20543581251404101 – Supplemental material for Outcomes of Patients With Autosomal Dominant Polycystic Kidney Disease Prescribed SGLT2 Inhibitors in British Columbia: A Single-Arm Retrospective Cohort Study Supplemental material, sj-pdf-1-cjk-10.1177_20543581251404101 for Outcomes of Patients With Autosomal Dominant Polycystic Kidney Disease Prescribed SGLT2 Inhibitors in British Columbia: A Single-Arm Retrospective Cohort Study by Alessandro Cau, Mark Elliott, Adeera Levin, Charith Karunarathna, Alexandra Romann, Ognjenka Djurdjev, Mohammad Atiquzzaman and Micheli Bevilacqua in Canadian Journal of Kidney Health and Disease
